# Chest wall TB and low 25-hidroxy-vitamin D levels in a 15-month-old girl

**DOI:** 10.1186/1824-7288-38-12

**Published:** 2012-04-17

**Authors:** Danilo Buonsenso, Benedetta Focarelli, Maria Scalzone, Antonio Chiaretti, Claudia Gioè, Manuela Ceccarelli, Piero Valentini

**Affiliations:** 1 Department of Pediatrics, Catholic University of the Sacred Heart - A. Gemelli Hospital, L.go A. Gemelli 8, 00168 Rome, Italy

## Abstract

Parietal chest wall tuberculosis is an extremely rare manifestation of tuberculosis (TB) in children. We present the case of a 15 month-old girl presenting with a chest wall lesion initially thought to be of neoplastic origin and eventually diagnosed as chest wall TB, which was treated with surgical debridement and specific antitubercular therapy. The girl had not-measurable 25-hidroxy-vitamin D levels, an increasingly recognized risk factor for the development of active TB. To our knowledge, in the English literature there are no similar described cases in such young infants. This case highlight the possibility of dealing with TB and its different manifestations also in low TB burden countries, due to continuously increasing migration flows. A detailed history is a key point to reach the diagnosis. Moreover, our case confirm the possible non casual relationship between TB and low 25-hidroxy-vitamin D levels, pointing out the importance of measuring its levels in all TB patients and considering its supplementation in addition to specific antitubercular therapy.

## Background

Tuberculosis (TB) of bones and joints accounts for about 10% of all extrapulmonary TB infections [1,2]. In developing countries, 1-5% of children less than 10 years of age with untreated primary infection due to *Mycobacterium tuberculosis *(Mtb) develop osteoarticular TB [1,3]. The spine is the most frequently affected site (40-60%), followed by methapyses of long bones, upper extremity bones and non-weight-bearing bones [3]. The infection reaches bones through a lymphohematogenous route, although direct spread from infected lymph nodes may occur [4]. Parietal chest wall TB is rare and rib TB is even rarer [5,6], constituting 1-3% of bone and joint TB [7].

Chest wall TB needs to be differentiate from benign and malignant tumors (chondroma, osteochondroma, fibrous dysplasia, lipoid granuloma, chondrosarcoma, myeloma multiplex) [8], metastatic carcinoma, lymphoma or other kinds of infection [9-12].

The diagnosis of chest wall TB is often delayed due to lack of specific symptoms and signs and an indolent course [13] and less than 50% of patients have a concomitant active pulmonary TB [8,14]. Radiologic findings vary depending on the stage at presentation [1,3]. Acid fast bacilli (AFB) and cultures of bone are positive in up to 75% of cases, and histopathology is often diagnostic [8].

The treatment of choice of chest wall TB is still debated, nevertheless the majority of described cases have been treated with surgical debridement (or excision based on lesion extension) and antitubercular therapy. Epidemiological and in vitro studies have found increasing evidences about a link between active TB and low 25-hidroxy-vitamin D (25-OH-D) levels, 25-OH-D replacement has been recently proposed as adjunctive therapy for TB treatment [15,16]. Nevertheless, the potential benefits of vitamin D as adjunctive therapy in mycobacterial infections are uncertain [17,18].

We present the case of a 15 month-old girl, with not-measurable 25-hidroxy-vitamin D (25-OH-D) levels, presenting with a chest wall lesion initially thought to be of neoplastic origin eventually diagnosed as chest wall TB, treated with surgical debridement and specific antitubercular therapy. At one year follow-up no disease recurrence have been reported. To our knowledge, in the English literature there are no similar described cases in such young infants.

## Case presentation

A 15 month-old girl was referred to the emergency room of our institution because of the appearance of a progressively increasing right painless parasternal swelling.

**Figure 1:**
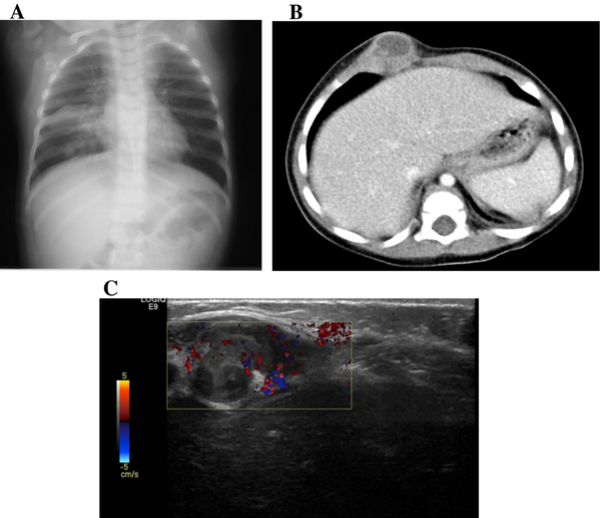
**a: chest x-Ray showing right lung hypodiaphany; b: Computed Tomography scan performed during the first hospitalization in Romania showing a ring-enhanced, well-circumscribed abscess adherent to the cartilagean rib; c: ultrasonography study performed at our institution, showing a lesion with peripheral area of hypervascularization**.

The girl had been admitted 2 weeks before in a hospital in Romania where chest wall ultrasonography (US), chest X-ray and chest computed tomography (CT) scan had been performed (Figure 1a, b). The US study revealed a well circumscribed, 25 mm per 9 mm in size, hypoechoic collection adherent to the cartilagean rib. Chest x-ray showed enlargement of hilar lymph nodes and right lung consolidation. Chest CT scan showed parenchymal consolidation with area of calcification in the right lower lobe, bilateral area of atelectasis, mediastinal and hilar lymph node enlargement, a patchy hypoechoic mass adjacent to the 10^th ^right cartilagean rib. Because of the association of chest wall swelling and lung lesion, a tumor was suspected and a bone marrow aspirate was performed, which ruled out neoplastic infiltration. A surgical management of both bone and parenchymal lesions was proposed by doctors, but parents refused and sought care at our hospital.

**Figure 2:**
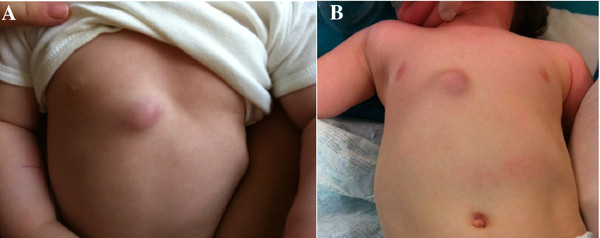
**a, b. The patient' lesion**.

On examination at our institution, the child had no fever, presented a cold, bluish, painless, right parasternal swelling (Figure 2). The physical examination was otherwise normal.

A detailed medical history was taken. The child had had two episodes of pneumonia when was 5 and 7 month-old, which were treated and cured with antibiotics and steroids. Family history was negative for neoplastic disease, but was positive for three cases of active TB. The child did not receive bacillus Calmette-Guérin vaccine at birth because it was not available. A new chest wall US study (Figure 1c) showed a well circumscribed, 25 mm per 12 mm in size, hypoechoic collection with peripheral area of hypervascularization adjacent to the cartilagean rib. The child was admitted at the Pediatric Infectious Disease Unit of our hospital.

**Figure 3:**
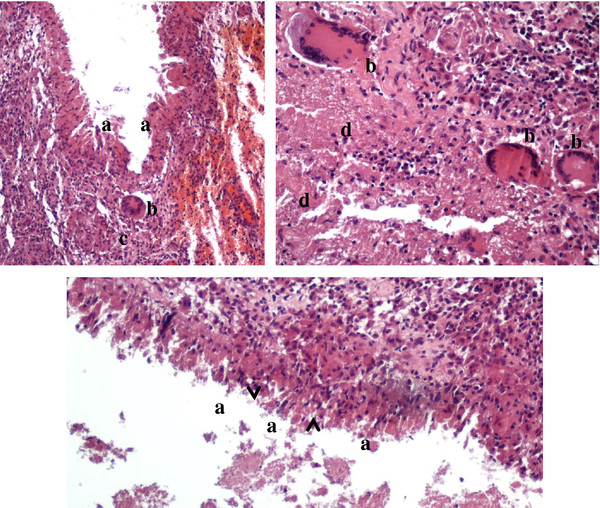
**a, b, c. Hystopathologic findings of the chest wall lesion showing typical findings of a tuberculosis infected tissue**. Hematoxylin- and eosin-stained sections. **a: **bands of epithelioid histiocytes (palisading arrangement); **b: **Langhans giant cells; **c: **discrete granulomas; **d: **caseation necrosis (acellular pink areas of necrosis with karyorrhectic debris). Kind concession of Vellone VG, Department of Pathology, Policlinico Gemelli, Catholic University, Rome, Italy.

Investigations revealed normal hemogram, liver and renal function tests, blood sugar and urinalysis, low levels of serum 25-OH-D (< 7 ng/ml) with normal calcium, phosphorus, PTH and alkaline phosphatasis level, normal serum protein electrophoresis, negative serology for human immunodeficiency virus. QuantiFERON TB Gold In Tube test (QTF) resulted positive (> 10 IU/l) and tuberculin skin test (TST) showed a 15 mm induration at 48 hours. Even though the use of QTF test in children is still unclear, we performed it since we are currently performing a correlation study between QTF and TST in children; nevertheless, in our case QTF result was only one of a number of elements (history, clinical and radiologic findings and TST result) which let us suspect a TB aetiology. Early morning gastric aspirates performed on three consecutive days were negative for acid-fast bacilli, but both Polymerase Chain Reaction (PCR) and culture for Mtb were positive. A surgical debridement of the lesion was performed. Hystopathologic studies revealed a predominantly lymphomononuclear infiltrate with epithelioid cells and Langhans giant cells (Figure 3). PCR and culture for Mtb were positive on pus and tissue smears. A diagnosis of chest wall and pulmonary TB was performed, antituberculous therapy (isoniazid, rifampin and pyrazinamide) was started and vitamin D was replaced with ergocalciferol 4000 IU daily, both for one year. During treatment, the patient had no adverse drug reactions and experienced no disease recurrence (based on clinical evaluation with detailed auxological evaluation and chest x-Ray studies - preferred to CT studies because of lower radiation burden, which showed resolution of pulmonary involvement and only a residual scar related to the surgery) at one year follow-up.

## Discussion

Tubercular parietal chest wall abscess is a rare form of extrapulmonary TB [19]. In a study, chest wall TB accounted for 1-5% of all cases of bone and joint TB [5]. Rib TB is rarely observed, occurring in 1-3% of all cases of bone and joint TB [12]. In Tatelman's series [20] rib TB was observed in 5% of all cases of bone and joint TB, and only in 0.1% of all hospital admissions for TB over a 10 year-period.

This rare localization of TB is more common in high TB burden countries and the majority of cases occur in children and young adults (range 4-76 years, mean age 25-30 years) [8].

TB rib lesions usually involve the midshaft of rib or the parasternal area [21]. The most frequent site of involvement described by Tatelman and Drouillard [20] was the rib shaft (61%), followed by the costovertebral joint (35%) and costochondral junction (13%). However, Johnson and Rothstein [22] and Lee et al. [23] found the costochondral junction to be the most frequent site of involvement. In agreement with the literature, our patient's lesion was adherent to the costochondral joint.

Associated pleuropulmonary TB, which was present in our case, has been reported in about 30 to 40% of cases [24].

Chest wall TB is believed to be attributed mainly to hematogenous dissemination, as we think it happened in our patient. Nevertheless, the presence of an underlying contiguous tuberculous mediastinitis, a lung cavity or a chest wall lymphadenitis can suggest local extension to the rib [25].

As with any diagnostic problem, careful evaluation of the clinical history and physical findings, together with any specific laboratory tests, may contribute considerably to the differential diagnosis.

The history of TB infection in the past or close contact with people with active TB is helpful to suggest the possibility of TB, though it is not always present. In this case, the patient had a family history of three close relatives with active TB, a data that should always let the physician to include TB in the differential diagnosis. Interestingly, TB was not considered in this case even in a high TB burden country like Romania.

Prolonged period of the presence of a swelling, cold abscess formation (the absence of which makes the diagnosis difficult [26]) and non-healing sinus are the common presentation.

In addition to history and a good clinical examination, use of radiologic studies (chest X-Ray, US and CT scan which can help demonstrate the skeletal lesion and any associated abscess or lung involvement [23]), bone scintigram, histologic and bacteriologic examination (using PCR which increases the diagnostic yield) of the operative specimen obtained by biopsy (whether open or CT guided, biopsy remains the gold standard for the diagnosis) are necessary steps to achieve a definitive diagnosis [5,27-29].

The differential diagnosis of chest wall lesions is wide and comprises mainly benign and malignant tumors and infections (table 1). Among the infective rib lesions, mycobacterial and bacterial infections are seen with equal frequency, followed by fungal ostemyelitis [30]. Apart from Mtb, bacterial infections caused by *Staphylococci, Streptococci, Salmonella, Haemophilus influenzae, Brucella *and *Actinomycetes*, and fungi infections such as coccidioidomycosis and blastomycosis can also cause rib osteomyelitis [30]. The benign tumors involving rib are chondroma (commonest), osteochondroma, fibrous dysplasia, and lipoid granuloma while the malignant lesions are chondrosarcoma, myeloma multiplex and secondary deposits from the lung and breast. All these present as expansion of the rib at the affected site, lytic lesion or pathological fractures [8]. In general, TB is the second most common cause of destructive rib lesions after metastatic neoplasms.

In our case, the origin in a high TB burden country and the presence of close relatives with active TB in the family, made us suspect about a possible lung TB with dissemination to the chest wall. Our suspicion was confirmed by a positive TST and Quantiferon TB-Gold in tube test, and by gastric washing microbiologic results. Once the diagnosis of lung TB was confirmed, we decided to manage surgically the chest wall lesion, which allowed us to have histopathological and microbiological confirmation of the TB atiology. Nevertheless, the best management of chest wall TB is still debated. A study of four cases stated that surgery is rarely indicated and antitubercular therapy is sufficient once the diagnosis is established by histology [5]. In another large series of 712 cases, spanning 11 years, surgical treatment was advocated for parietal chest wall TB [31]. Chen et al. also suggested that surgical debridement is necessary in treating TB of ribs followed by antitubercular therapy for six months [32]. Agrawal et al. recently described their experience with 7 patients (aged 4 to 18 years) with chest wall TB, which were all managed with surgery (either resection or curettage of the abscess cavity and extraction of the infected granulation tissue) followed by one-year antitubercular therapy. No relapses were observed after 5 to 8 years of follow-up [8]. In agreement with Agrawal and Chen experiences, we also support surgical management (either resection or curettage, based on operative findings) as it ensures the removal of the diseased area, enables histopathological confirmation, enhances post-surgical neovascularization in the healing tissues which aids in better distribution of the antitubercular drugs, thereby increasing the efficacy and ultimate response to therapy [8].

We chose a three drug regimen for our patient instead of using 4 drugs for the first 2 months of treatment, since a three drug regimen has a proven 99% cure rate [33]; quadruple therapy with the addition of ethambutol or streptomycin in the intensive phase is generally recommended where there is high risk of isoniazid-drug resistance [33], which we considered was not the case for our patient. The good treatment response of our patient and no relapse at one year follow-up prove that the chosen treatment was adequate for this case.

Our patient had non measurable levels of 25-OH-D. The Endocrine Society's clinical practice guidelines define vitamin D deficiency as 25-OH-D < 20 ng/ml (50 nmol/l) and vitamin D insufficiency as 25-OH-D levels of 21-29 ng/ml (52.5-72.5 nmol/l) [34]. This aspect is of particular interest due to recent findings on the role of 25-OH- D in the modulation of the immune response and the epidemiological correlation between low 25-OH- D levels and TB.

In fact, clinical outcome of TB depends primarily on cell-mediated innate and acquired immune responses, where macrophages and T lymphocytes play an essential role. The first step of infection after transmission of Mtb into the lower respiratory tract involves the activation of macrophages, the primary host cells for mycobacteria, via toll-like receptors. Toll-like receptor-mediated activation of human macrophages by Mtb upregulates expression of the genes encoding VDR and vitamin-D-1-hydroxylase (CP27B), the enzyme that converts 25-hydroxycholecalciferol (25OHD) to calcitriol (1,25(OH)2D). This leads to a vitamin D-dependent production of LL-37, an antimicrobial peptide of the cathelicidin family, within macrophages and increased macrophage-dependent killing of mycobacteria. Additionally, cathelicidin modulates the immune response to Mtb by attracting peripheral blood neutrophils, monocytes, and T cells [17,35].

An association between TB and vitamin D deficiency is suggested by numerous observational studies. A meta-analysis examined 3 prospective studies and 4 case-control studies published between 1980 and 2006 and found a medium-to-strong association between low serum values and the risk of active TB in humans [15]. In addition, Martineau et al. [16] discover a striking temporal relationship between vitamin D deficiency and TB. The reporting of new TB cases in Cape Town, South Africa, was lowest in the months after the seasonal increase in serum 25-OH-D levels, whereas the reporting of new TB cases was highest in the months following the season with the lowest serum 25-OH-D levels [36]. Nevertheless, the potential benefits of vitamin D as adjunctive therapy in mycobacterial infections are uncertain. A randomized, double-blind, placebo-controlled trial in a large cohort of TB contacts in London indicated that a single dose of 2.5 mg vitamin D had an interferon-γ independent growth restrictive effect on mycobacteria [37]. A systematic review concluded that evidence do not clearly support the use of 25-OH-D in the treatment of TB but supports further research into its use as adjunctive TB therapy [17,18].

In conclusion, our report describes a rare TB localization in a very young children, the youngest described in the English literature. This case highlight the possibility of dealing with TB and its different manifestations also in low TB burden countries, due to continuously increasing migration flows. A detailed history is a key point to reach the diagnosis. Moreover, our case confirm the possible non casual relationship between TB and low 25-OH-D levels, pointing out the importance of performing clinical trials aimed to better understand the role of 25-OH-D in TB pathogenesis and its use in addition to specific antitubercular therapy for the treatment of TB.

## Consent

Written informed consent was obtained from the child's parent for publication of this report and any accompanying images.

## Ethical approval

This report was approved by the University's Institutional Review Board and Ethical Commette.

## Competing interests

The authors declare that they have no competing interests.

## Authors' contributions

PV made a substantial contribution in conception and design and critically revised the manuscript. DB and PV acquired, analyzed and interpreted the data and drafted the manuscript. MS, MC and CG contributed to the acquisition of data. DB and BF analyzed the data and critically revised the manuscript. AC made a contribution in conception of the manuscript. All authors gave final approval of the version to be published.

## Sources of funding

None
